# A deep image-to-image network organ segmentation algorithm for radiation treatment planning: principles and evaluation

**DOI:** 10.1186/s13014-022-02102-6

**Published:** 2022-07-22

**Authors:** Sebastian Marschner, Manasi Datarb, Aurélie Gaasch, Zhoubing Xu, Sasa Grbic, Guillaume Chabin, Bernhard Geiger, Julian Rosenman, Stefanie Corradini, Maximilian Niyazi, Tobias Heimann, Christian Möhler, Fernando Vega, Claus Belka, Christian Thieke

**Affiliations:** 1 Department of Radiation Oncology, University Hospital, LMU Munich, Munich, Germany; 2 Technology Excellence, Digital Technology & Innovation, Siemens Healthineers, Erlangen, Germany; 3 Technology Excellence, Digital Technology & Innovation, Siemens Healthineers, Princeton, NJ USA; 4 Department of Radiation Oncology, University of North Carolina at Chapel Hill, Chapel Hill, NC USA; 5 Cancer Therapy, Siemens Healthineers, Forchheim, Germany; 6 Department of Radiation Oncology, LMU Klinikum, Marchioninistr. 15, 81377 München, Germany

**Keywords:** Radiation, Planning, Algorithm, Deep-image-to-image-network, Contour, Organs at risk, Thorax, Pelvis

## Abstract

**Background:**

We describe and evaluate a deep network algorithm which automatically contours organs at risk in the thorax and pelvis on computed tomography (CT) images for radiation treatment planning.

**Methods:**

The algorithm identifies the region of interest (ROI) automatically by detecting anatomical landmarks around the specific organs using a deep reinforcement learning technique. The segmentation is restricted to this ROI and performed by a deep image-to-image network (DI2IN) based on a convolutional encoder-decoder architecture combined with multi-level feature concatenation. The algorithm is commercially available in the medical products “syngo.via RT Image Suite VB50” and “AI-Rad Companion Organs RT VA20” (Siemens Healthineers). For evaluation, thoracic CT images of 237 patients and pelvic CT images of 102 patients were manually contoured following the Radiation Therapy Oncology Group (RTOG) guidelines and compared to the DI2IN results using metrics for volume, overlap and distance, e.g., Dice Similarity Coefficient (DSC) and Hausdorff Distance (HD_95_). The contours were also compared visually slice by slice.

**Results:**

We observed high correlations between automatic and manual contours. The best results were obtained for the lungs (DSC 0.97, HD_95_ 2.7 mm/2.9 mm for left/right lung), followed by heart (DSC 0.92, HD_95_ 4.4 mm), bladder (DSC 0.88, HD_95_ 6.7 mm) and rectum (DSC 0.79, HD_95_ 10.8 mm). Visual inspection showed excellent agreements with some exceptions for heart and rectum.

**Conclusions:**

The DI2IN algorithm automatically generated contours for organs at risk close to those by a human expert, making the contouring step in radiation treatment planning simpler and faster. Few cases still required manual corrections, mainly for heart and rectum.

## Introduction

In radiation treatment planning, delineating the target volumes and organs at risk (OAR) is one of the most important and time-consuming tasks. The dose-volume histogram analysis for plan evaluation, contour-based visual guidance in image-guided radiation therapy, and the dose-response assessment of radiation side effects are depending on the accuracy of the delineation [1, 2]. The contouring workload will further increase due to more widely used strategies of adaptive planning where the treatment plan is adapted to anatomical changes such as tumor shrinkage, requiring re-contouring during the fractionated treatment course.

The need for fast and accurate delineation has led to a variety of automated computer-based approaches. Most autosegmentation algorithms used in clinical practice today are atlas-based and required sophisticated atlas creation of self-made contours, with difficulties in case of anatomical variations, low contrast organs or when the anatomy is modified by the presence of a tumor. In addition, atlas-based segmentation is computationally intensive and can take several minutes to complete [3–5]. In the last years, artificial intelligence (AI) and machine learning approaches were developed for autosegmentation aiming to improve accuracy and shorten the time needed for segmentation even in complex anatomical situations [6–8].

In this work, we describe and evaluate an AI algorithm for autosegmentation of organs at risk based on a deep image-to-image-network (DI2IN).

## Materials and methods

### Autosegmentation with a deep image-to-image network (DI2IN)

**Fig. 1:**
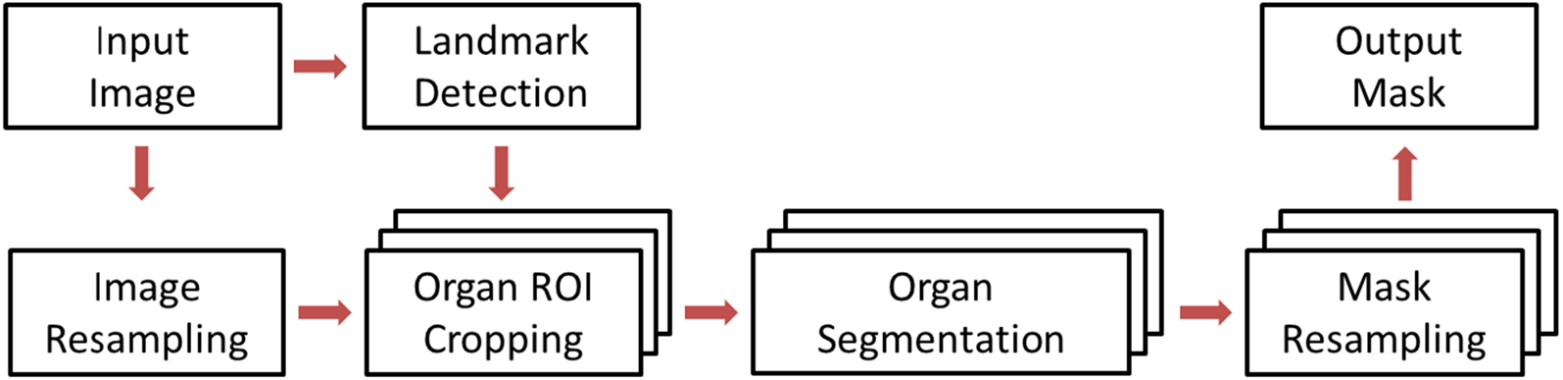
Autosegmentation data flow. Illustration of the autosegmentation data flow with landmark detection for ROI definition, image resampling, organ segmentation and mask resampling

The general data flow of the autosegmentation algorithm provided by SIEMENS Healthineers is illustrated in Fig. 1. The input image is the computed tomography (CT) image of the patient in full resolution. Image resampling is applied as normalization to acquire an isotropic image to present the realistic aspect ratio of the anatomical structures. Due to the high complexity and thus resource requirement from the organ segmentation model, the image is typically downsampled to reduce the computational burden of the algorithm execution.

The automatic segmentation is performed on the region of interest (ROI) around individual organs or a group of multiple organs instead of the entire image volume. This helps the segmentation model to focus on capturing variations from the organs themselves without disruptions of irrelevant structures and significantly reduces the computational resources. The anatomical landmark detection was trained independently from segmentation algorithms with manually annotated landmark points across the human body as described by Ghesu et al. [9]. To locate the ROIs, anatomical landmarks (including vessel bifurcations, bony structures, and organ center and boundary points) are detected using a deep reinforcement learning technique [9] from the input image. For each landmark, an agent is trained to search for the best path to walk towards the landmark from any location of the image. Specifically, considering the current state as an image patch centred at the current voxel, the agent learns to take one of the actions from the current voxel so that the distance towards the landmark of interest is minimized. During testing time, the agent will move one step at a time and eventually stop at or around the desired landmark where the action estimation converges. To reduce the computational costs, a multi-stage strategy is integrated to search the landmark position at different scales of the image resolution, where the action at the coarser resolution will move the agent close to the landmark position with effectively larger step size. Given the detected landmarks and their heuristic relationships with the organs, ROIs are cropped with the sizes derived from training data distribution with its center based on the associated landmark position and its size being large enough to cover the organs to be segmented.

**Fig. 2:**
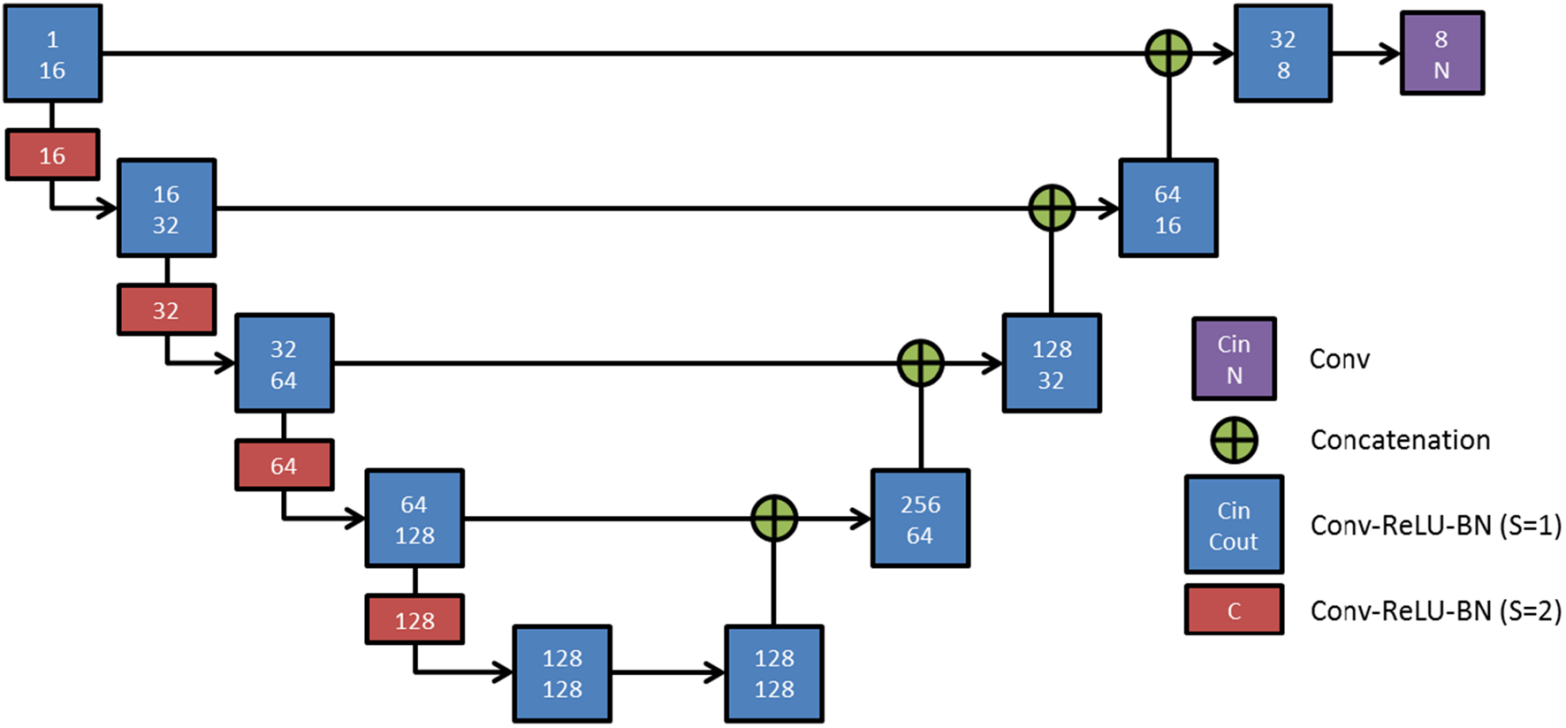
Deep Image-to-Image Network (DI2IN) for organ segmentation. Deep Image-to-Image Network (DI2IN) for organ segmentation. S: stride, Conv: convolution, Cin: number of input channels, Cout: number of output channels, C: number of channels for convolutions where the input and output channels are equal, ReLU: rectified linear unit, BN: batch normalization, N: number of output channels, N is set to 1 for single-organ segmentation, and to 1 + number of organs for multi-organ segmentation

A deep image-to-image network (DI2IN) [10] based on a convolutional encoder-decoder architecture combined with multi-level feature concatenation is employed for the automatic segmentation step (Fig. 2). Compared to traditional U-Net [6], additional convolutional layers with stride of 2 (red blocks in Fig. 2) are used in the encoder of DI2IN instead of max pooling layers to increase the receptive field while reducing sizes of feature maps. In the decoder of DI2IN, trilinear interpolation is used to upsample the activation maps back to the original input image size. During the training process, the network was driven by a cross-entropy loss based on a learning rate of 0.001 using the ADAM [11] optimization. The algorithm was trained for the segmentation of left and right lung (using 10,000 cases), heart (386 cases), and bladder and rectum including anus and rectosigmoid flexure (784 cases). The training CT data were collected from multiple hospital sites. Data annotation was performed based on RTOG guidelines by a team trained with anatomical knowledge and mentored by radiologists and radiation oncologists.

**Fig. 3:**
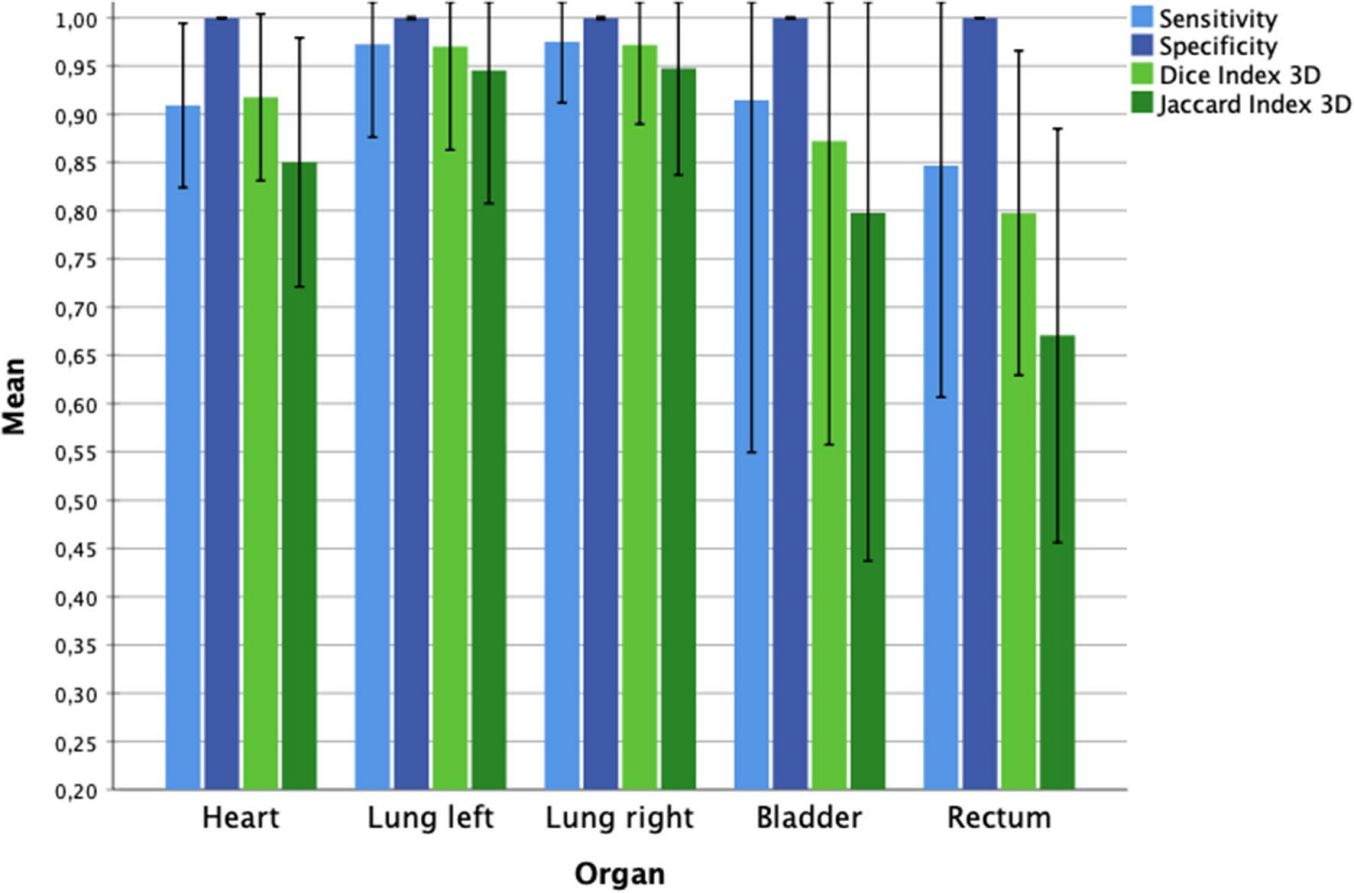
Overlap measurements. Overlap measurements of manual and automated contours. Mean values and standard deviation

**Fig. 4:**
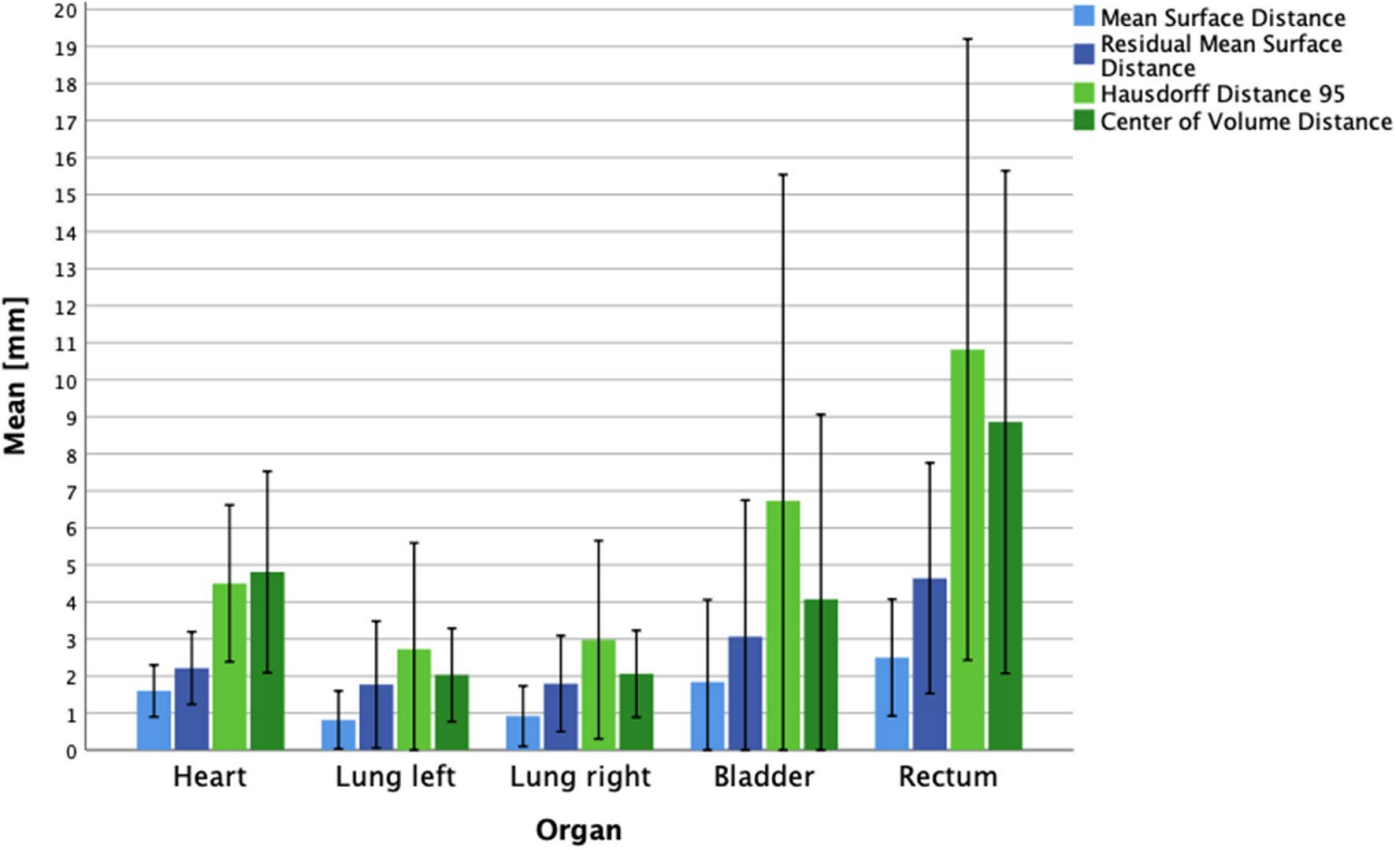
Distance measurements. Distance measurements between manual and automated contours. Mean values and standard deviation

After organ segmentation, the estimated organs mask is resampled back to the original image resolution, where each organ-specific mask is aggregated in a single multi-organ mask.

### Evaluation patient cohort

An independent evaluation patient cohort was established from CT images of patients treated at LMU (Ludwig-Maximilians-Universität) university hospital. The scans were acquired for treatment planning without contrast medium on a Toshiba CT scanner with 3 mm slice thickness. For the thoracic region, 237 female patients treated for breast cancer were included, resulting in 237 usable heart contours and 233/234 usable left/right lung contours. For the pelvic region, 102 male and female patients treated for various tumors (e.g. cervical and prostate cancer) were included resulting in 98 usable bladder and 102 usable rectum contours. OARs with gross tumor volume or tumor infiltration were excluded.

The CT data was anonymized for the scientific purpose of this work. This study complies with the declaration of Helsinki, Good Clinical Practice (GCP) and Good Epidemiological Practice (GEP). The data acquisition and analysis were in accordance with Bavarian hospital law (Art.27 Abs. 4 BayKrG).

### Manual segmentation

All manual contours were drawn by an experienced radiation oncologist following the guidelines of the RTOG [12, 13] using Oncentra Masterplan by Elekta AB, Sweden. *Lungs*: All inflated and collapsed, fibrotic and emphysematic lungs were contoured including small vessels extending beyond the hilar regions; hilars and trachea/main bronchus were not included. *Heart*: Contoured along the pericardial sac. The superior aspect (base) began at the level of the inferior aspect of the pulmonary artery passing the midline and extend inferiorly to the apex of the heart. *Bladder*: Contoured inferiorly from its base, and superiorly to the dome. *Rectum*: Contouring ended inferiorly from the lowest level of the ischial tuberosities (right or left), and superiorly before the rectum lost its round shape in the axial plane and connected anteriorly with the sigmoid.

### Comparison of manual and automatic segmentation

Manual contours (MAN) were considered as ground truth and were compared to the automatic contours (AUTO) generated by a software prototype (provided by Siemens Healthineers) of the DI2IN algorithm. We used several quantitative geometric measures in the categories volume (absolute and ratio), overlap (Sensitivity, Specificity [14], Jaccard Conformity Index [15, 16], Dice Similarity Coefficient [17], Discordance Index [18, 19], Geographical Miss Index) and distance (Mean Surface Distance [14], Center of Volume Distance, Residual Mean Surface Distance [20, 21], Hausdorff Distance HD_95_ [22], and difference of the superior, inferior, right, left, anterior and posterior boundaries defined by the furthest reaching voxel belonging to the contour in the respective direction). All formulas used are summarized in the appendix. All results were imported into IBM SPSS Statistics version 25.0.0 and subsequently processed and analysed.

Additionally, MAN and AUTO were inspected visually for identification of regions which are still challenging for the algorithm.

## Results

### General algorithm performance

In all cases, the DI2IN algorithm was able to generate the automatic contours without any user interaction. The computation with the prototype took roughly 30 s per organ.

### Volume comparison

The volumes of manual (MAN) and automatic (AUTO) contours are summarized in Table 1. The highest variations in absolute volume from patient to patient were observed for the left and right lung as the largest structure types, however, the mean volume difference between manual and automatic contours e.g. of the left lung were only 17 ml. Also, for all other organs the absolute volumes of manual and automatic contours were similar, with the volume ratio either 0.9 or 1.0.

**Table 1:** Volume comparison

	Bladder mean ± SD	Rectum mean ± SD	Heart mean ± SD	Lung left mean ± SD	Lung right mean ± SD
Volume MAN [ml]	331 ± 178	89 ± 39	569 ± 115	1,897 ± 604	2,220 ± 647
Volume AUTO [ml]	362 ± 209	97 ± 44	557 ± 108	1,914 ± 623	2,240 ± 668
Difference (MAN-AUTO) [ml]	32 ± 74	8.1 ± 22	11 ± 54	17 ± 158	19 ± 151
Ratio (MAN/AUTO)	0.9 ± 0.2	0.9 ± 0.2	1.0 ± 0.1	1.0 ± 0.1	1.0 ± 0.1

Volume comparison between manual (MAN) and automatic contours (AUTO), mean values ± standard deviation

### Overlap and distance measurements

The results for all metrics of overlap and distance comparisons are summarized in Table 2.

**Table 2:** Overlap and distance measurements

	Bladder mean ± SD	Rectum mean ± SD	Heart mean ± SD	Lung left mean ± SD	Lung right mean ± SD
Sensitivity	0.93 ± 0.15	0.84 ± 0.1	0.91 ± 0.04	0.98 ± 0.03	0.98 ± 0.03
Specificity	0.99 ± 0.01	0.99 ± 0.01	0.99 ± 0.01	0.99 ± 0.01	0.99 ± 0.01
Jaccard Conf. Index (JCI) 3D	0.81 ± 0.15	0.67 ± 0.10	0.85 ± 0.06	0.95 ± 0.06	0.95 ± 0.05
Dice Sim. Coeff. (DSC) 3D	0.88 ± 0.13	0.79 ± 0.08	0.92 ± 0.04	0.97 ± 0.05	0.97 ± 0.04
Discordance Index (Disl) 3D	0.13 ± 0.1	0.22 ± 0.10	0.06 ± 0.07	0.03 ± 0.05	0.03 ± 0.05
Geographical Miss Index 3D	0.07 ± 0.15	0.16 ± 0.12	0.08 ± 0.04	0.02 ± 0.03	0.03 ± 0.03
Center of volume comp. [mm]	4.1 ± 5.0	8.9 ± 6.8	4.7 ± 2.6	2.0 ± 1.3	2.1 ± 1.7
Mean surface dist. (MSD) [mm]	1.8 ± 2.2	2.5 ± 1.6	1.6 ± 0.7	0.8 ± 0.8	1.0 ± 0.8
Residual mean surface dist. (RMSD) [mm]	3.1 ± 3.7	4.6 ± 3.1	2.2 ± 0.93	1.8 ± 1.7	1.8 ± 1.3
Hausdorff Distance (HD_95_) [mm]	6.7 ± 8.8	10.8 ± 8.4	4.4 ± 2.0	2.7 ± 2.7	2.9 ± 2.7
Right Boundary [mm]	2.9 ± 4.7	− 0.9 ± 4.1	0.0 ± 1.4	− 4.9 ± 19.2	0.8 ± 1.2
Left Boundary [mm]	0.1 ± 3.7	2.5 ± 16.1	− 0.3 ± 1.8	− 0.26 ± 1.0	2.5 ± 6.0
Anterior Boundary [mm]	2.0 ± 5.1	5.2 ± 10.5	− 0.4 ± 2.1	0.6 ± 1.5	0.7 ± 1.6
Posterior Boundary [mm]	− 1.9 ± 11.7	1.7 ± 5.8	0.0 ± 2.0	− 0.1 ± 2.2	− 0.2 ± 1.9
Inferior Boundary [mm]	− 0.3 ± 4.8	7.0 ± 13.2	− 8.5 ± 4.8	− 1.8 ± 4.1	− 1.5 ± 3.3
Superior Boundary [mm]	− 1.8 ± 10.6	− 8.7 ± 16.9	− 4.9 ± 8.7	− 0.7 ± 2.0	− 0.5 ± 1.6

Overlap and distance measurements between manual and automatic contours as mean values ± standard deviation (SD)

The overlap measurements sensitivity, specificity, Jaccard Conformity Index (JCI) and Dice Similarity Coefficient (DSC) are illustrated in Fig. 3. The sensitivity showed values of 0.98 for the left/right lungs, 0.93 for the bladder and 0.91 for the heart, and the lowest value for the rectum with 0.84. The specificity was excellent with 0.99 for all structure types. The JCI with mean values of 0.95 for the right and left lung showed nearly complete overlap between manual and automatic contours. Again, the poorest result was obtained for the rectum with a JCI of 0.67. The same ranking was seen for the DSC, with best values for left/right lung (DSC 0.97), then heart (DSC 0.92), bladder (DSC 0.88) and rectum (DSC 0.79).

The Discordance Index (DisI) performed best for left and right lung with only 3% of AUTO being outside MAN. Heart, bladder and rectum reach 6%, 13% and 22%, respectively. Comparable results were seen for the Geographical Miss Index (GMI), where for the left/right lungs 2/3% of MAN were outside AUTO, 8% for the bladder, 7% for the heart, and 16% for the rectum.

The distance measurements in terms of center of volume distance, MSD, RMSD and HD_95_ are illustrated in Fig. 4. The MSD and RMSD showed mean values between 0.8 and 4.6 mm for all organs. The Hausdorff distance HD_95_ was best for left /right lung with of 2.7/2.9 mm, and worst for the rectum with 10.8 mm

Most boundary differences where around or below 3 mm (the thickness of one CT slice). Bigger discrepancies were seen for the superior, inferior and anterior boundaries of the rectum with − 8.7 mm, 7.0 mm and 5.2 mm respectively, and the inferior and superior boundary of the heart with a mean deviation of − 8.5 mm and − 4.9 mm respectively.

**Fig. 5:**
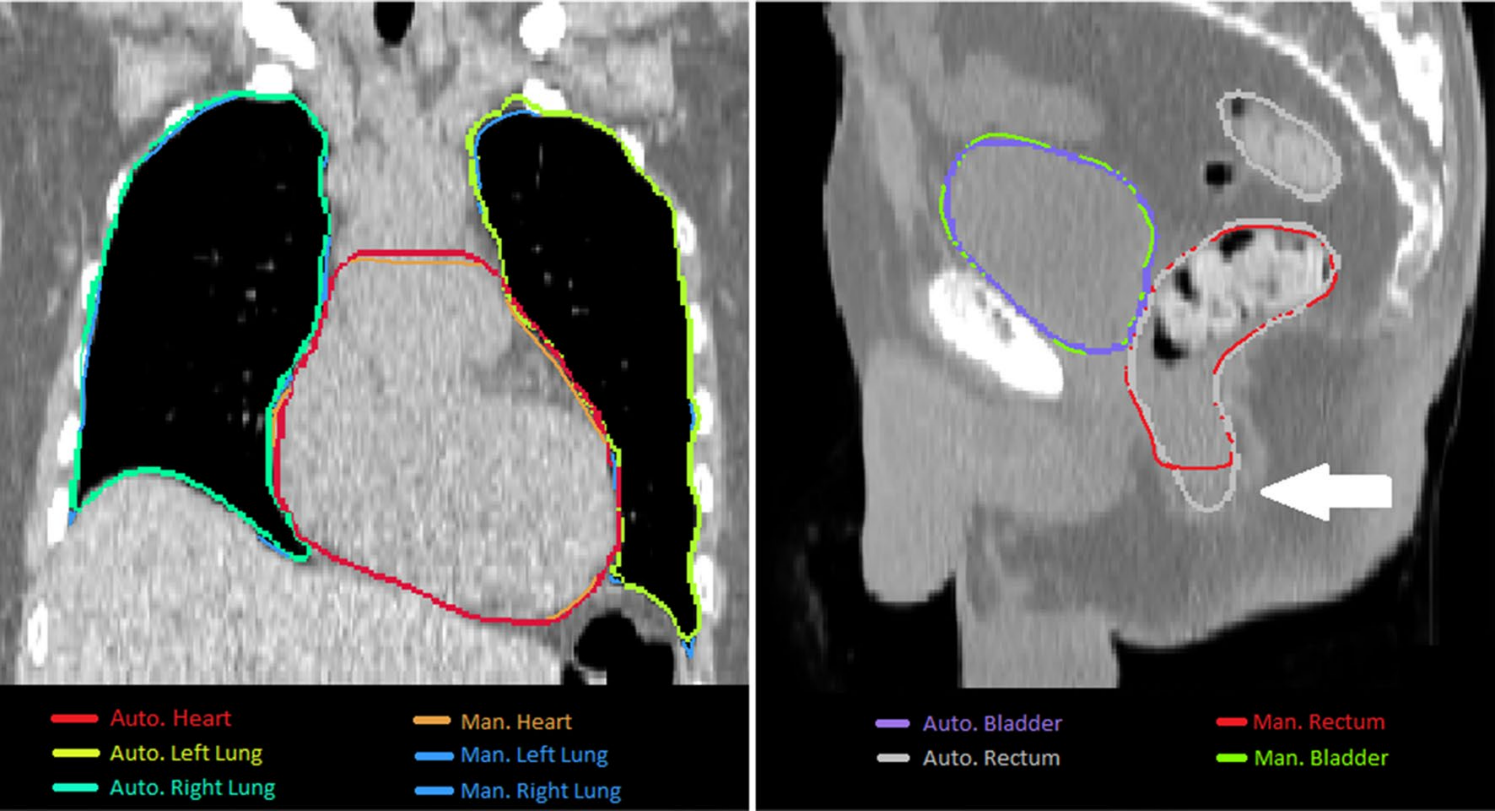
Exemplary cases. Left panel: Exemplary case with manual and automated contours of left and right lung and heart. Right panel: Exemplary case with manual and automated contours of rectum and bladder. The arrow indicates the difference at the inferior boundary of the rectum

The visual inspection showed an overall excellent agreement between manual and automated contours, with the most challenging organs, as already identified by the boundary analysis, being the rectum and the heart. Two exemplary cases are shown in Fig. 5.

## Discussion

The quantitative comparison between MAN and AUTO contours showed an excellent agreement in most cases for all geometric metrics in terms of volume, overlap and distance, with some exceptions especially for the heart and the rectum. The discrepancies for the rectum can be explained by the fact that the training data set did include anus and the rectosigmoid flexure, but the manual segmentation did not. Some cases would require manual corrections before the automatic contours could be used for radiotherapy planning, however even in these cases the time needed by a human expert for editing the autogenerated organ contours would likely be less than generating the complete contour manually from scratch.

Delpon et al. [23] compared five commercial atlas-based segmentation software solutions for radiation planning to manual segmentation for 10 patients for bladder and rectum: ABAS (Elekta Oncology Systems, Crawley, UK), WorkFlow Box (Mirada Medical Ltd., Oxford, UK), MIM Maestro (MIM Software Inc., USA), SPICE (Philips N.V., Netherlands) and RayStation (RaySearch Laboratories AB, Sweden). For the rectum, the software systems achieved a volume ratio of 0.9–1.3 (this study: 0.9) and a mean DSC of 0.49–0.75 (this study: 0.79). For the bladder, the atlas-based software systems achieved a volume ratio of 1.01–1.62 (this study: 0.9) and a mean DSC of 0.62–0.81 (this study: 0.88). In another study using the atlas-based system ABAS, Kim et al. reported DSC values for the bladder below 0.6 [24]. These findings indicate that the algorithm presented in this work is able to outperform atlas-based algorithms for organ segmentation.

In recent years, a significant focus was put on the development and evaluation of machine learning based algorithms. Feng et al. [25] developed a deep convolutional neural network for autosegmentation of thoracic organs and reported DSC 0.972/0.979 and HD_95_ 2.103/3.958 mm for left/right lung. Regarding these metrics, our algorithm achieved an almost identical performance (DSC 0.97/0.97, HD_95_ of 2.7/2.9 mm). Cardenas et al. reported in their review about deep learning autosegmentation architecture types [26] mean DSC values between 0.89 and 0.93 for the heart, 0.93–0.98 for the lungs and 0.7–0.84 for bladder. In comparison, the algorithm presented here achieved equivalent results for the heart (DSC 0.92) and right/left lung (DSC 0.97), and superior results for the bladder (DSC 0.88). Considering that the evaluations were not performed on the same data sets, we conclude that the accuracy of the algorithm presented here is at least comparable to other modern machine and deep learning-based algorithms.

Sultana et al. [27] used a two-step hierarchical convolutional neural network segmentation strategy for automatic contouring of multiple organs of the pelvis, combining an UNet architecture with a generative adversarial network. They reported excellent mean DSC values for bladder of 0.95 (this study: 0.88) and rectum of 0.90 (this study: 0.79), however based on a single center and relatively small cohort of 290 training and 15 test cases.

Lustberg et al. [28] compared a prototype version of the “Mirada DLC Expert” (Mirada Medical Ltd., Oxford, UK), which utilizes convolutional neural networks, to the atlas-based autosegmentation “WorkFlow Box” of the same company and to manual segmentation, and found that the DLC expert showed promising results for automatically generating high quality contours, providing a greater time saving compared to existing solutions.

We consider the comparably large unseen evaluation cohort size of 237 patients for the thorax region and 102 patients for the pelvic region to be a strength of this study. We also aimed at a comprehensive geometric evaluation to facilitate the comparison with other studies. A limitation is that for the thorax region only female patients were used. We assume that the findings are transferable to male patients, however this needs to be confirmed by further studies and by thorough inspection in clinical practice. Another limitation is that only one human reader was used. Multiple human readers would allow the assessment of inter-observer variations, which can be quite substantial [14, 29, 30]. For another algorithm it has already been shown that the accuracy of deep-learning based autosegmentation is comparable to inter-observer variability [31]. It can be speculated that automatic algorithms might even become able to contour organs at risk with a higher reproducibility and accuracy than humans, especially when less experienced readers are included [1, 2, 32, 33]. This could, in addition to the time savings, also increase the quality of the radiation treatment planning, like it has been discussed for head and neck patients in [34].

The algorithm in the software prototype used in this study corresponds to the algorithm implemented in two products by Siemens Healthineers (Erlangen, Germany), the server-based “syngo.via RT Image Suite” (version VB50) and the cloud-based “AI-Rad Companion Organs RT” (version VA20), for lungs, rectum and bladder. The heart model evaluated in this study is slightly improved with regard to the latest released product versions at the time of submission of this publication. Both products are commercially available and are certified for clinical use, making this study relevant for clinical practice.

As future work it is planned to include multiple human readers to assess inter-observer variations and an analysis of the dosimetric consequences of contour differences as a metric with more direct clinical impact than geometric parameters.

## Conclusions

We described and evaluated a commercially available deep image-to-image network (DI2IN) algorithm for automatic contouring of organs at risk in radiation treatment planning. The automatic contours showed excellent agreements with manual contours drawn by an experienced radiation oncologist, with some deviations mostly at the lower part of the heart and both upper and lower parts of the rectum.

## Appendix

The metrics used in this work were calculated as follows:

$$Sensitivity=\frac{AUTO \cap MAN}{MAN}$$(from [14]).

$$Specificity= \frac{\stackrel{-}{AUTO} \cap \stackrel{-}{MAN}}{\stackrel{-}{MAN}}$$ (from [14]).

*(* $$\stackrel{-}{AUTO}=Volume outside AUTO, \stackrel{-}{MAN}=Volume outside MAN)$$

$$Jaccard Index \left(JCI\right)=\frac{AUTO \cap MAN}{AUTO \cup MAN}$$(from [15, 16])

$$Discordance Index \left(DisI\right)=1-\left(\frac{MAN\cap AUTO}{AUTO}\right)$$ (from [18, 19])

$$Dice Similarity Coefficient= \frac{2 (AUTO\cap MAN) }{AUTO+MAN}$$(from [17])

$$Surface Distance \left(DSM\right)= \frac{1}{{n}_{s}+{n}_{{s}^{{\prime }}}}\left(\sum _{p=1}^{{n}_{s}}d\left(p,{S}^{{\prime }}\right)+ \sum _{{p}^{{\prime }}=1}^{{n}_{{S}^{{\prime }}}}d\left({p}^{{\prime }}, S\right)\right)$$ (from [14])

$$Residual Mean Surface Distance \left(RMS\right)=\sqrt{\frac{1}{{n}_{s}+{n}_{{s}^{{\prime }}}}\left(\sum _{p=1}^{{n}_{s}}d{\left(p,{S}^{{\prime }}\right)}^{2}+ \sum _{{p}^{{\prime }}=1}^{{n}_{{S}^{{\prime }}}}d{\left({p}^{{\prime}},\text{S}\right)}^{2}\right)}$$ (from [20, 21])

$$Hausdorff Distance \left(HD\right)=max \left[d\left(S,{S}^{{\prime }}\right), d({S}^{{\prime }}, S)\right]$$ (from [22])

## Data Availability

The datasets used and/or analysed during the current study are available from the corresponding author on reasonable request.
