# Correction: A deep image-to-image network organ segmentation algorithm for radiation treatment planning: principles and evaluation

**DOI:** 10.1186/s13014-022-02110-6

**Published:** 2022-08-23

**Authors:** Sebastian Marschner, Manasi Datar, Aurélie Gaasch, Zhoubing Xu, Sasa Grbic, Guillaume Chabin, Bernhard Geiger, Julian Rosenman, Stefanie Corradini, Maximilian Niyazi, Tobias Heimann, Christian Möhler, Fernando Vega, Claus Belka, Christian Thieke

**Affiliations:** 1 Department of Radiation Oncology, University Hospital, LMU Munich, Munich, Germany; 2 Technology Excellence, Digital Technology & Innovation, Siemens Healthineers, Erlangen, Germany; 3 Technology Excellence, Digital Technology & Innovation, Siemens Healthineers, Princeton, NJ USA; 4 Department of Radiation Oncology, University of North Carolina at Chapel Hill, Chapel Hill, NC USA; 5 Cancer Therapy, Siemens Healthineers, Forchheim, Germany; 6 Department of Radiation Oncology, LMU Klinikum, Marchioninistr. 15, 81377 München, Germany

## Correction: Radiation Oncology (2022) 17:129 https://doi.org/10.1186/s13014-022-02102-6

After publication of this article [1], the authors reported that the author name ‘Manasi Datar’ was incorrectly written as ‘Manasi Datarb’.

The original article [1] has been corrected.
